# Efficiency above 12% for 1 cm^2^ Flexible Organic Solar Cells with Ag/Cu Grid Transparent Conducting Electrode

**DOI:** 10.1002/advs.201901490

**Published:** 2019-09-30

**Authors:** Yunfei Han, Xiaolian Chen, Junfeng Wei, Guoqi Ji, Chen Wang, Wenchao Zhao, Junqi Lai, Wusong Zha, Zerui Li, Lingpeng Yan, Huiming Gu, Qun Luo, Qi Chen, Liwei Chen, Jianhui Hou, Wenming Su, Chang‐Qi Ma

**Affiliations:** ^1^ School of Nano‐Tech and Nano‐Bionics University of Science and Technology of China Hefei 230027 P. R. China; ^2^ Suzhou Institute of Nano‐Tech and Nano‐Bionics Chinese Academy of Sciences (CAS) Collaborative Innovation Center of Suzhou Nano Science and Technology Suzhou 215123 P. R. China; ^3^ Institute of Chemistry Chinese Academy of Sciences Beijing 100190 P. R. China

**Keywords:** 1 cm^2^, Ag/Cu grid, flexible organic solar cells, large‐area

## Abstract

With the rapid progress of organic solar cells (OSCs), improvement in the efficiency of large‐area flexible OSCs (>1 cm^2^) is crucial for real applications. However, the development of the large‐area flexible OSCs severely lags behind the growth of the small‐area OSCs, with the electrical loss due to the large sheet resistance of the electrode being a main reason. Herein, a high conductive and high transparent Ag/Cu composite grid with sheet resistance <1 Ω sq^−1^ and an average visible light transparency of 84% is produced as the transparent conducting electrode of flexible OSCs. Based on this Ag/Cu composite grid electrode, a high efficiency of 12.26% for 1 cm^2^ flexible OSCs is achieved. The performances of large‐area flexible OSCs also reach 7.79% (4 cm^2^) and 7.35% (9 cm^2^), respectively, which are much higher than those of the control devices with conventional flexible indium tin oxide electrodes. Surface planarization using highly conductive PEDOT:PSS and modification of the ZnO buffer layer by zirconium acetylacetonate (ZrAcac) are two necessary steps to achieve high performance. The flexible OSCs employing Ag/Cu grid have excellent mechanical bending resistance, maintaining high performance after bending at a radius of 2 mm.

## Graphical abstract

**High performance flexible organic solar cells with efficiency** above 12% for 1 cm^2^ cells are fabricated using a Ag/Cu composite grid electrode. The excellent optical and electrical properties of the Ag/Cu electrode contribute to the high performance and good mechanical resistance of the flexible organic solar cell.

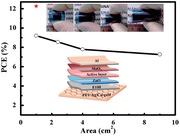

Organic solar cells (OSCs) have received considerable attention and have demonstrated great potential as flexible, lightweight, transparent, and low‐cost energy sources due to their intrinsic lightweight and flexible properties. Recently, because of the rapid developments in the synthesis of new materials,^1^, ^2^, ^3^ optimization of the active layer morphology,^4^, ^5^ and interface engineering,^6^, ^7^, ^8^, ^9^ the power conversion efficiencies (PCEs) of OSCs have exceeded 16%^10^ and 17%^11^ for single and tandem solar cells, respectively. However, all these high‐ efficiency OSCs are laboratory‐ scale devices, mostly with effective areas smaller than 0.1 cm^2^. The development of large‐area OSCs (area >1 cm^2^) severely lags behind the growth of small‐area OSCs, especially those with flexible substrates. Though flexible OSCs have been greatly developed,^12^ and efficiencies over 10% have been achieved using flexible indium tin oxide (ITO),^13^ silver nanowires,^14^, ^15^ metal grid,^16^, ^17^ and conductive polymer electrodes,^18^, ^19^ most of these works, including that obtaining the highest efficiency of 12.55% were carried out with and reported for cells around 0.04 cm^2^.^13^ As the device area is scaled up, the performance suffers dramatic drop. As summarized by Wei and co‐workers,^20^ the highest PCE of large‐area single junction flexible OSCs with an area of 1 cm^2^ is only ≈8%,^21^, ^22^ obviously lower than the record for small‐area rigid cells. In terms of the transition from mature laboratory‐scale small‐area coating technology to scalable of thin film photovoltaics, the performance of cells exceeding 1 cm^2^ in size is often considered as the critical value.^6^, ^21^, ^23^, ^24^, ^25^, ^26^ Thus, further improvements in the performance of large‐area (>1 cm^2^) cells are desired for practical applications of flexible OSCs in the future energy market.

The low efficiency of large‐area flexible OSCs is partly due to the conductivity of the flexible transparent conducting electrode, which is one of the essential components of flexible OSCs. In flexible OSCs, commercial ITO is the most widely used transparent conductive film for the electrode.^12^ Though it has superior photoelectric performances when used in small‐area rigid OSCs, ITO has several intrinsic drawbacks regarding its use in high performance large‐area flexible OSCs, i.e., brittleness, high sheet resistance, and low infrared transmittance. To address the brittleness issue, several transparent conducting materials, such as carbon nanotubes,^27^, ^28^, ^29^ graphene,^30^, ^31^ metallic nanowires,^32^, ^33^ conducting polymers,^18^, ^34^ and thin metals^35^ have been used as substitutes for flexible ITO. These novel electrodes exhibited excellent mechanical properties that enabled the flexible OSCs to be sufficiently robust to withstand repeated bending^33^, ^36^, ^37^ or even stretching.^34^ However, these electrodes are still problematic when applied to highly efficient large‐area OSCs because of their low electrical conductivity and transparency in the visible range. For the scaling‐up of flexible OSCs, a large sheet resistance of the transparent conducting electrode will cause serious electrical loss,^38^ resulting in a dramatic drop in the performance parameters for large‐area OSCs, particularly the short circuit current density (*J*_SC_) and fill factor (FF).^39^, ^40^, ^41^, ^42^ Since this electrical loss in the large‐area photovoltaic cells highly depends on the sheet resistance of the electrodes, many efforts have been made to improve the electrode conductivity.^43^ The conventional approaches to improve the conductance of the transparent electrode generally cause a negative effect because of lowering the optical transparency. Therefore, an important goal for transparent electrodes would be achieving a reasonable balance between optical and electrical properties. The metal rigid electrode exhibited extremely low sheet resistance compared to other transparent conducting electrodes and excellent optical transparency.^44^, ^45^, ^46^ Thus, better large‐area performance might be expected using this electrode.

In this work, we present a flexible Ag/Cu composite grid electrode that contains high‐resolution hexagonal silver and copper grids on 120 µm thick poly(ethylene terephthalate) (PET) substrates. The thickness of the Ag/Cu composite grid is only 3 µm and most of the region is blank; thus, such an electrode shows high transparency in the visible light region, with an average transmittance ≈84%. Additionally, the 3 µm thick Ag/Cu composite grid is almost embedded in the PET substrate. The large aspect ratio^47^, ^48^ (depth‐ to‐width ratio, 1:1) endows this electrode with a relatively low sheet resistance, less than 1 Ω sq^−1^. To the best of our knowledge, this sheet resistance is among the lowest values for a transparent conducting electrode.^49^ Aiming to achieve high‐ efficiency large‐area flexible OSCs, we carefully optimized 1 cm^2^ flexible OSCs based on this Ag/Cu composite grid electrode, and achieved a highest efficiency of 12.26%, which is the highest efficiency for 1 cm^2^ scale flexible OSCs to date. The certificated efficiency is 11.45%. Large‐area flexible OSCs of 4 and 9 cm^2^ exhibit PCEs of 7.79% and 7.35%, respectively. Surface planarization is the critical factor for achieving high performance for these electrode‐based cells. A 215 nm thick E100 layer (a type of high conductively PEDOT:PSS) was incorporated with the Ag/Cu grid to form a composite hybrid; with this process, the typical short circuit was suppressed and the charge transfer ability was improved, consequently leading to a significantly enhanced performance. The flexible OSCs showed excellent bending resistance, retaining 95% of the initial efficiency after 2000 bending cycles. This work showed the importance of transparent electrode with high transmittance and conductivity for large‐area flexible OSCs, and provided a practical interface modification approach to achieve high efficiency and long‐term stability for large‐area flexible OSCs. These results would be important and meaningful to the module design and furthers commercialization of large‐area flexible OSCs.

**Figure** **1**a shows a photograph and a schematic diagram of the large‐area PET/Ag/Cu grid electrode. This electrode composed of a periodical hexagonal Ag/Cu grid with a grid width of 3 µm and a grid spacing of 194 µm. The 2.5 µm thick Ag grid was embedded in the PET substrate through nanoimprinting, and the 500 nm thick Cu grid was deposited on top of the Ag grid through electroplating.^47^, ^50^ Herein, the modification of the Ag grid by electroplated Cu on one side can fill the groove that existed in the imprinted Ag grid, leading to a smoother and more uniform surface, and on the other side can improve the electrical conductivity. The schematic diagram of the fabrication process is illustrated in Figure S1 of the Supporting Information. It contains five steps: making the Ni master plate, patterning the PET substrate through imprinting, filling the Ag nanoinks into the trenches through doctor blading, electroplating the Cu grid on top of the Ag grid, and finally polishing (see the Experimental Section for more information). For the grid electrode with a fixed pattern, the thickness of the Ag/Cu grid will affect the conductivity, while the grid width and grid spacing will influence the optical transmission. The coverage of the substrates by the metal grid is only ≈3% (Figure S2, Supporting Information), thereby leading to quite low optical loss. Such a low optical loss results in a high optical transmission for this flexible electrode (85–90%), which is only around 3% lower than transmittance of the PET substrate (for the transmittance spectra of the PET substrate, see Figure S3, Supporting Information). Characterization of the flexible electrode was first investigated, and the transmittance spectra and conductivity of the PET/Ag/Cu grid, glass/ITO, and PET/ITO electrodes are shown in Figure 1b. The PET/Ag/Cu grid electrodes exhibit improved optical transmission in the region of 300–600 nm in comparison with the conventional PET/ITO electrode. According to these transmittance curves, the average transmittance in the wavelength region of 300–1100 nm, defined as an average of the transmission, can be calculated by dividing the total transmittance by the total number of data points. The calculated values for the PET/Ag/Cu grid, glass/ITO and PET/ITO electrode are 84.5%, 83.9%, and 79.2%, respectively. Obviously, the PET/Ag/Cu grid electrode shows higher optical transparency than the conventional PET/ITO electrode, which might enable more light harvesting in the photoactive layer and lead to higher *J*_SC_. In addition to better optical transparency, the PET/Ag/Cu grid electrode also shows an outstanding advantage in terms of the electrical conductivity. The sheet resistance of this electrode is less than 1 Ω sq^−1^, which is one order of magnitude lower than those of conventional rigid and flexible ITO electrodes, and is the best conductivity in comparison with other flexible transparent electrodes.^49^ We know that the electrical conductivity of an ITO electrode relies on the thickness and the crystallization quality of the ITO films.^51^ Though a lower sheet resistance would be expected upon increasing the thickness of the ITO films, a thicker ITO film would inevitably decrease the optical transparency.^52^ In the meanwhile, a thicker ITO film on the PET substrates still has not contributed to a better conductivity, which was attributed to poorer crystallization of ITO because of lower temperature annealing (below 200 °C).^53^, ^54^ In terms of the overall optical and electrical properties, the PET/Ag/Cu grid film has great potential as a transparent flexible electrode. The evolution of sheet resistance (Figure S4a, Supporting Information) showed slight increase of sheet resistance from 0.62 ± 0.10 to 0.88 ± 0.19 Ω sq^−1^ after 260 h storage in air (with temperature of 25 ± 5 °C and humidity of 80% ± 10%), which should be attributed to the oxidation of Cu and Ag (Figure S4b, Supporting Information). However, the sheet resistance of the electrode still kept around 1Ω sq^−1^, and tended to be stable after exposure 200 h in air, indicating the electrode oxidation mainly occurred at the first stage around 200 h. The further oxidation of electrode might be passivated due to the dense microstructure of the surface Cu layer that fabricated through electroplating. For the use in organic solar cells, notably the surface of the PET/Ag/Cu grid electrode is not flat, with an ≈180 nm step between the metal rigid and the blank substrate; therefore, surface planarization is necessary before cell fabrication. Herein, the highly conductive PEDOT:PSS (named E100) was used to modify the Cu/Ag composite electrode. The thickness of the E100 layer was controlled by regulating the concentration of the E100 aqueous and the spin‐coating speed. Figure 1c of the Supporting Information shows the relationship between the E100 thickness and the transmittance and conductivity of the Ag/Cu/E100 composite electrodes. With increasing the E100 thickness from 100 to 380 nm, the average transmittance of the PET/Ag/Cu/E100 composite electrode gradually decreases to 80.5% 78.2%, and 76.7%. This result is consistent with the simulated results (shown in Figure S5, Supporting Information). Such a decrease in the transmission is due to the optical absorption of E100 from 300 to 1100 nm. However, the sheet resistance of the Ag/Cu/E100 electrodes remained at ≈1 Ω sq^−1^, even when the thickness of E100 layer is 380 nm. As shown in Figure S6 of the Supporting Information, the sheet resistance of the E100 film is about 4.5, 1.3, and 0.9 kΩ sq^−1^ for the 100, 215, and 380 nm thick E100 film. Therefore, the high conductivity of the PET/Ag/Cu/E100 composite electrode even with a thick E100 layer could be due to the excellent conductivity of Ag/Cu grid and the reasonable conductivity of E100. All the transmittance and sheet resistance of these electrodes are listed in Table S1 of the Supporting Information. According to the sheet resistance (*R*_s_) and transmittance at 550 nm (*T*), the Haacke figure of merits (FoM), which is widely used to evaluate the comprehensive photoelectronic properties of transparent electrodes,^55^, ^56^ can be calculated using the equation *Φ*_TC_ = *T* ^10^/*R*_s_.^57^ As shown in Table S1 of the Supporting Information, the PET/Ag/Cu, and PET/Ag/Cu/E100 electrodes exhibit a *Φ*_TC_ of (≈1–3.2) × 10^−1^ Ω^−1^, which is much higher than those of both the rigid (2.61 × 10^−2^ Ω^−1^) and flexible (3.19 × 10^−3^ Ω^−1^) ITO electrodes.

**Figure 1:**
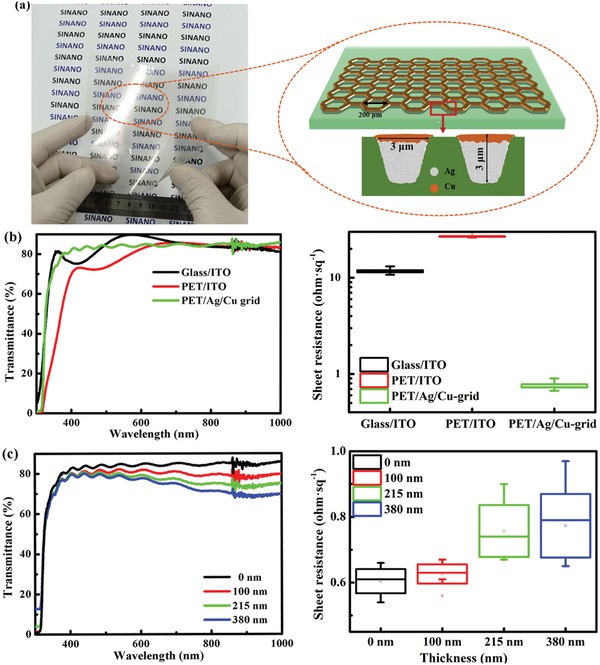
a) Photograph and the schematic diagram of the large‐area PET/Ag/Cu electrode. b) Transmittance spectra and sheet resistance of the glass/ITO, PET/ITO, and PET/Ag/Cu electrodes. c) Transmittance spectra and sheet resistance of PET/Ag/Cu/E100 composite electrodes with different E100 thicknesses.

With the PET/Ag/Cu/E100 flexible electrode, a series of inverted architecture flexible devices were fabricated. **Figure** **2**a shows the device structure of the flexible OSCs based on the Ag/Cu composite electrode, and as well as the molecular structure of the organic donors and acceptors. First, the photovoltaic performances of the flexible PBDB‐T:ITIC solar cells with different E100 thicknesses were compared. Rigid and flexible solar cells with ITO electrodes were also fabricated as references. Figure 2b shows the *J*–*V* characteristics of these flexible cells, and the corresponding performance parameters are listed in **Table** **1**. We observed that the pristine Ag/Cu electrode without any modification could not work well in flexible OSCs despite the excellent electrode properties. This result is reasonable since the large step in the PET/Ag/Cu electrode would cause direct contact of the metal grid electrode and the top Al, and consequently resulting in device short circuit. With the modification of the Ag/Cu grid electrode by the E100 layer, the PBDB‐T:ITIC cells exhibited a reasonable device performance. Additionally, the thickness of E100 also greatly influences the device performance. With a relatively thin E100 layer (<100 nm), the Ag/Cu grid electrode cannot be covered well, and direct contact of the Ag/Cu grid electrode with the evaporated Al top electrode would cause leakage. Regarding the performance parameters, the device fill factor (FF) is much lower than that of the other devices, which is caused by the low shunt resistance (Table S2, Supporting Information). When the thickness of the E100 layer is increased to ≈215 nm, the 1 cm^2^ flexible OSCs exhibit a reasonable PCE above 9%. However, further increases in the E100 thickness to 380 nm leads to a performance decrease to 8.5%. The reduced performance caused by the thicker E100 layer could be due to inferior charge transfer at the cathode. Based on these results, the optimized thickness of the E100 layer would be around 220 nm. With such modification, the optimized performance of 1 cm^2^ PBDB‐T:ITIC solar cells is 9.54%, which is comparable to and higher than those of rigid and flexible ITO‐based cells, respectively. To investigate the applicability of this flexible electrode to high performance fullerene and nonfullerene OSCs, flexible PTB7‐Th:PC_71_BM and PBDB‐TF:IT‐4F cells were also fabricated. Unlike the PBDB‐T:ITIC solar cells, the PTB7‐Th:PC_71_BM and PBDB‐TF:IT‐4F flexible solar cells with the same structure show relatively low performance, which is mainly due to their poor FFs. Such poor FFs (60% for the PTB7‐Th:PC_71_BM cell, and 60% for the PBDB‐TF:IT‐4F cell) might be due to the insufficient electron extraction between the acceptor and the ZnO cathode buffer layer. Therefore, a thin zirconium acetylacetonate (ZrAcac) layer was inserted between the ZnO buffer layer and the active layer through simply spin‐coating the chloroform dissolved ZrAcac solution. Previously, ZrAcac has been used as the cathode buffer layer to modify the cathode in organic and perovskite solar cells.^58^, ^59^, ^60^, ^61^ A thin ZrAcac layer with proper thickness would form interface dipole, reduce trap states, and thus reduce charge transport resistance.^60^ Herein, the insertion of ZrAcac between ZnO and the active layer led to increase of shunt resistance for the PTB7‐Th:PC_71_BM and PBDB‐TF:IT‐4F cells (Table S2, Supporting Information). Consequently, FF of the PTB7‐ThPC_71_BM and PBDB‐TF:IT‐4F cells improved to 63%, and 67%, respectively. And as a consequence, the 1 cm^2^ flexible PTB7‐ThPC_71_BM and PBDB‐TF:IT‐4F solar cells gave a champion performance of 8.15%, and 11.18%, respectively. The PBDB:ITIC device with ZnO/ZrAcac also gave a reasonable performance of 9.37%. In order to achieve higher performance, 1 cm^2^ flexible solar cells with NF3000‐P:NF3000‐N active layer were fabricated. NF3000‐P and NF3000‐N are the polymer donor and small‐molecular acceptor, respectively. With the same interface modification approach, the cell gives a highest performance of 12.26%. Notably, the efficiency above 12% is the efficiency record for the large‐area flexible OSCs to the best of our knowledge.^7^, ^42^, ^62^, ^63^ The *J*–*V* characteristics and external quantum efficiency (EQE) spectra of the NF3000‐P:NF3000‐N flexible OSCs were measured by the third‐party, and the curves are showed in Figure 2d,e. The certification report is showed in the Supporting Information, and a certified PCE of 11.45% is acquired. Herein the certified PCE is an average value obtained from three times *J*–*V* sweeping both from forward and backward scan. An average *J*_SC_ of 19.48 mA cm^−2^ is observed from *J*–*V* sweeping. But we found the integrated *J*_SC_ determined from EQE spectra by the third‐party (as shown in Figure S7, Supporting Information) is ≈20.73 mA cm^−2^. Thus, a higher certification performance of 12.22% can be achieved if using the integrated *J*_SC_. Figure 2f shows the performance histogram of 1 cm^2^ PTB7‐Th:PC_71_BM, PBDB‐T:ITIC, PBDB‐TF:IT‐4F, and NF3000‐P:NF3000‐N flexible OSCs calculated from 12 to 15 devices. All the heterojunction solar cells exhibit good reproducibility, with at least 60% of devices giving PCEs exceeding 7.5%, 8.7%, 10.5%, and 11.5% for the PTB7‐Th:PC_71_BM, PBDB‐T:ITIC, PBDB‐TF:IT‐4F, and NF‐3000P:NF‐3000N cells. Figure 2g shows a comparison of the device performance presented in this work with the reported values. This figure shows that the PCEs of the flexible OSCs of both 1 cm^2^, as well as 4 and 9 cm^2^ (vide infra) reported in this work are the highest among previous reports.

**Figure 2:**
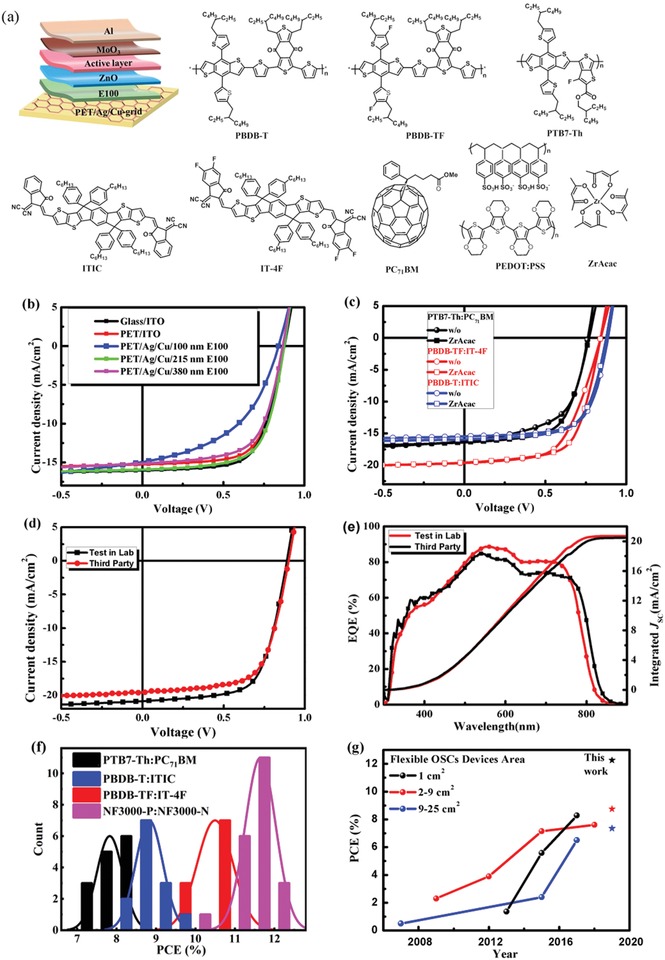
a) Device structure and the molecular structure of the organic semiconductor. b) *J*–*V* characteristics the 1 cm^2^ PBDB‐T:ITIC devices with glass/ITO, PET/ITO, and PET/Ag/Cu/E100 electrodes. c) *J*–*V* characteristics of the 1 cm^2^ PTB7‐Th:PC_71_BM, PBDB‐TF:IT‐4F, and NF3000‐P:NF3000‐N devices. d) *J*–*V* characteristics of the NF3000‐P:NF3000‐N cells. e) EQE spectra of the NF3000‐P:NF3000‐N cells. f) Histogram of the PCEs. g) Comparison of the performance of the device presented in this work with those of previously reported flexible OSCs.^21^, ^22^, ^64^, ^65^, ^66^, ^67^, ^68^

**Table 1:** Device performance of the 1 cm^2^ PBDB‐T:ITIC, PTB7‐Th:PC_71_BM, PBDB‐TF:IT‐4F, NF3000‐P:NF3000‐N solar cells with different electrodes

Entry	Active layer	Electrode	E100 thickness [nm]	Cathode buffer layer	*V*_oc_ [V]	*J*_sc_ [mA cm^−2^]	FF [%]	PCE [%]	
								Best	Ave.^a)^
1	PBDB‐T:ITIC	Glass/ITO	0	ZnO	0.88	16.04	66	9.32	9.16 ± 0.27
2	PBDB‐T:ITIC	PET/ITO	0	ZnO	0.88	15.26	66	8.86	8.57 ± 0.42
3	PBDB‐T:ITIC	PET/Ag/Cu/E100	0	ZnO	–	–	–	–	–
4	PBDB‐T:ITIC	PET/Ag/Cu/E100	100	ZnO	0.86	14.23	47	5.75	5.70 ± 0.07
5	PBDB‐T:ITIC	PET/Ag/Cu/E100	215	ZnO	0.88	15.94	68	9.54	8.83 ± 0.35
6	PBDB‐T:ITIC	PET/Ag/Cu/E100	380	ZnO	0.87	15.19	64	8.46	8.40 ± 0.05
7	PBDB‐T:ITIC	PET/Ag/Cu/E100	215	ZnO/ZrAcac	0.89	15.48	68	9.37	9.01 ± 0.27
8	PTB7‐Th:PC_71_BM	PET/Ag/Cu/E100	215	ZnO	0.78	16.23	60	7.60	7.12 ± 0.35
9	PTB7‐Th: PC_71_BM	PET/Ag/Cu/E100	215	ZnO/ZrAcac	0.78	16.58	63	8.15	8.01 ± 0.03
10	PBDB‐TF:IT‐4F	PET/Ag/Cu/E100	215	ZnO	0.84	19.63	60	9.89	9.33 ± 0.51
11	PBDB‐TF:IT‐4F	PET/Ag/Cu/E100	215	ZnO/ZrAcac	0.84	19.87	67	11.18	10.44 ± 0.41
12	NF3000‐P:NF3000‐N	PET/Ag/Cu/E100	215	ZnO/ZrAcac	0.89	20.87	66	12.26	11.65 ± 0.39

^a)^ Average performance calculated over 10–15 individual devices.

Atomic force microscopy (AFM) images of the PET/Ag/Cu electrode, PET/Ag/Cu/E100, PET/Ag/Cu/E100/ZnO, and PET/Ag/Cu/E100/ZnO/active layer are shown in **Figure** **3**a–d. Both the surface roughness and the height differences between the Ag/Cu grid and blank PET region can be observed in these images. The step height shown in the schematic diagram in Figure 3c named as *d*‐value. A 150 nm high step occurs between the Ag/Cu grid and the blank PET substrate in the pristine Ag/Cu grid electrode, which means that the Ag/Cu grid is bossy in the flexible substrate. After depositing the E100 layer and E100/ZnO layers, this step height gradually decreased to 90 and 40 nm, respectively. We also tested the thicknesses of E100 and ZnO films on top of the glass substrates, and found that they were 210 and 80 nm, respectively. Suppose that the thicknesses of the E100 and ZnO layers on the PET substrate are the same to thicknesses on the glass substrates; then, the real thicknesses of E100 and ZnO on the Ag/Cu grid region can be estimated on the basis of the step height (*d‐*value). As a consequence, the real thicknesses of E100 and ZnO on the grid region are ≈150 and 40 nm, which are much thinner than the film on top of the blank PET substrate. This result suggests that both the E100 and ZnO films have different coverages on top of the metal and PET regions. However, interestingly, the step height remains at ≈40 nm after the deposition of the active layer, indicating that the organic photoactive layer has the same thin film thickness along entire electrode substrate. Based on the above observations, a schematic diagram was drawn to show the coverage on the PET and metal grid regions by the various layers in Figure 3e. Furthermore, cross‐section scanning electron microscopy (SEM) images of the PET/Ag/Cu electrode and the flexible PBDB‐T:ITIC solar cells were obtained and are presented in Figure 3f,g. A 180 nm high step between the metal grid and PET is observed in Figure 3f, proving that the surface of the Ag/Cu grid electrode is not flat. This observation is consistent with that of the AFM images. In Figure 3g, we clearly observe the sequential layers of Ag, Cu, E100, ZnO, PBDB‐T:ITIC active layer, MoO_3_, and Al from bottom to top. The thickness of each layer can be determined from the cross‐section view SEM images. In the Ag/Cu grid region, the layer thicknesses of the Ag, Cu, E100, ZnO, active layer, MoO_3_, and Al are determined to be about 2.5 µm, 500, 200, 55, 80, 18, and 220 nm. In the blank PET region, the thicknesses of the active layer, MoO_3_, and Al layers are identical to those in the Ag/Cu grid region. Nevertheless, the E100 layer in the blank PET region is 250 nm thick, which is 50 nm thicker than that in the Ag/Cu region. The thickness of the ZnO layer in PET region is slightly thicker than that in the Ag/Cu region. The different thicknesses of the E100 layer on the PET and metal grid region definitely prove the E100 layer was selectively deposited on the Ag/Cu grid and the blank PET, in good agreement with the AFM results. This result can be mostly attributed to the different wettability and contact properties of E100 on top of the metal and PET substrates. However, the main functional layers of the devices, i.e., ZnO, active layer, MoO_3_, and Al electrode have identical thicknesses on the PET and metal grid regions.

**Figure 3:**
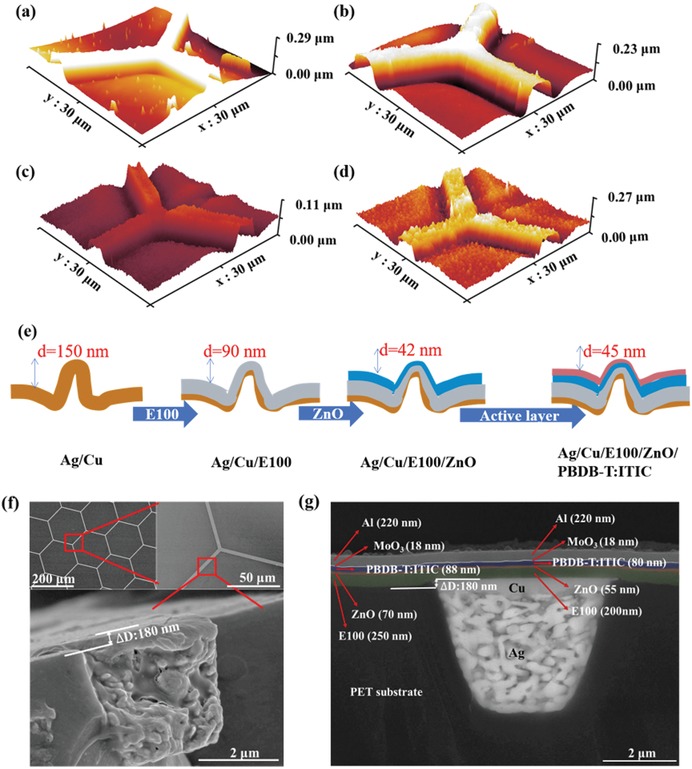
AFM images of a) PET/Ag/Cu electrode, b) PET/Ag/Cu/E100, c) PET/Ag/Cu/E100/ZnO, and d) PET/Ag/Cu/E100/ZnO/PBDB‐T:ITIC. e) Schematic diagram of the deposition process of the flexible OSCs on top of the PET/Ag/Cu substrate. f) SEM and cross‐section SEM images of the blank PET substrate and g) cross‐section SEM images of the flexible PBDB‐T:ITIC device.

Next, we evaluated the mechanical durability and the long‐term stability of the flexible PBDB‐T:ITIC OSCs. As shown in **Figure** **4**a, the flexible OSCs were first manually bent around pillars of different radii (*r* = ∞,10, 7.5, 5, 3.5, and 2 mm) for 20 bending cycles. Figure 4b–e shows the blending resistance of the PBDB‐T:ITIC OSCs under different bending radii, and the flexible cells with the PET/ITO electrode were also investigated for comparison. The PET/Ag/Cu flexible cells maintained nearly the same performance as the initial value after bending at radii of 10, 7.5, and 5 mm, and no meaningful change was found. For the bending radii of 3.5 and 2 mm, the PCEs of the Ag/Cu grid electrode‐based cells are still 93% and 82% of the initial performance. By contrast, the performance of the flexible ITO‐based solar cells dramatically decreased after bending at radii of 5 and 3.5 mm. Such poor mechanical durability of the ITO‐based device has already been reported, and has been readily ascribed to the formation of cracks in the ITO electrode.^69^ Due to the formation of cracks (Figure S8, Supporting Information), a largely increased sheet resistance from 26 to ≈450 Ω sq^−1^ is observed for the flexible ITO electrode (Figure S9, Supporting Information). By contrast, the sheet resistance of the PET/Ag/Cu grid electrode almost kept constant (Figure S9, Supporting Information). We also investigated the evolution of the performance after 2000 bending cycles at a bending radius of 7.5 mm. The efficiency of the Ag/Cu grid electrode‐based devices slightly decreases from 8.72% to 8.26%, remaining at 95% of the initial value after 2000 bending cycles. For the flexible PET/ITO devices, however, the PCE continuously decreases as the number of bending cycles increasing. After 200 bending cycles, the devices almost cannot work. The superior mechanical robustness of the Ag/Cu grid electrode devices indicates that such flexible OSCs are suitable for roll‐to‐roll fabrication. Figure 4g shows the performance evolution of the unencapsulated flexible OSC devices during long‐term storage in the N_2_‐filled glove box. As illustrated in this figure, the 1 cm^2^ PBDB‐T:ITIC and PBDB‐TF:IT‐4F flexible OSCs show performances of 9.6%, and 7.58% after 6 months, which are 90% (initial efficiency: 10.63%) and 87% (initial efficiency: 8.71%) PCEs of the initial values, respectively.

**Figure 4:**
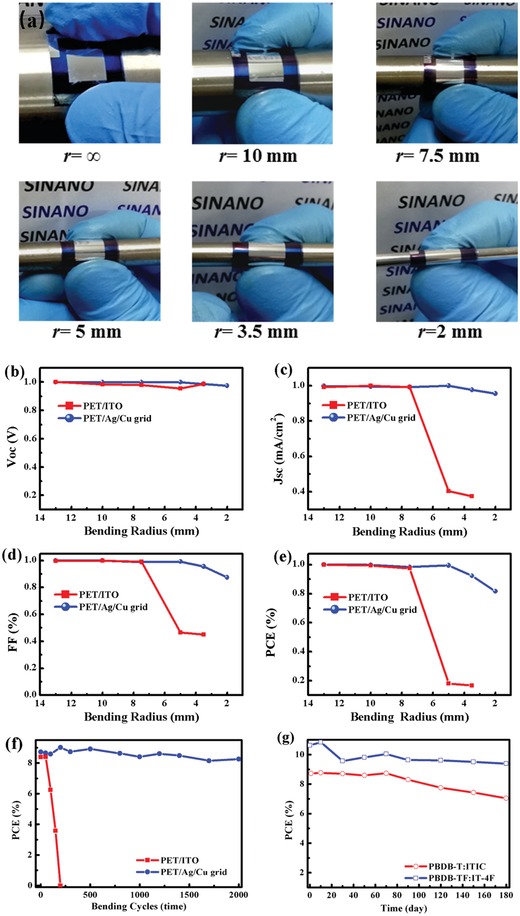
a) Photographs of the devices. Evolution of b) *V*_OC_, c) *J*_SC_, d) FF, and e) PCE during bending with different bending radii. f) The PCE decay of the flexible OSCs with PET/ITO and PET/Ag/Cu grid electrodes. g) Evolution of the performance during long‐term storage in the N_2_ glove box.

Using this Cu/Ag/E100 composite electrode, large‐area flexible OSCs based on the PBDB‐T:ITIC active layer were also fabricated. **Figure** **5** shows the *J*–*V* characteristics and the EQE spectra of these devices. The performance parameters are listed in **Table** **2**. For the 2.4 cm^2^ cells, optimized device performances and average PCE are 8.75% and 8.29%, respectively. For the 4 and 9 cm^2^ cells, optimized performances of 7.79% and 7.35% are obtained, which are nearly the highest values as far as we know. For comparison, flexible large‐area PBDB‐T:ITIC cells on PET/ITO with the same area were studied and presented an optimized performance of 6.61% (2.4 cm^2^) and 5.88% (4 cm^2^), respectively. This result clearly demonstrates an overwhelming advantage of the PET/Ag/Cu grid electrode for use in large‐area high efficiency flexible OSCs. Nevertheless, the device performance, particularly the FF still gradually decreases with increasing for both the Ag/Cu grid and flexible ITO electrode‐based cells. To obtain deep insight into the reduced FF, the *J*–*V* simulation was carried out using an equivalent circuit model.^40^ The simulated *J*–*V* characteristics and performance parameters were showed in Figure S10 and Table S3 of the Supporting Information. As exhibited by the simulation results, a dramatic decline performance is observed for the flexible ITO‐based cells, while the performance of the Ag/Cu‐based cells shows only a slight decrease. This observation again demonstrates the great advantage of the Ag/Cu grid for high performance large‐area cells. In the aspect of lower FF for the experimental result compared to the simulated value for the Ag/Cu‐based cells, it might be ascribed to more films defects and inferior interface contact in large‐area films.^68^ Based on this result, improved performance can be speculated through optimizing the film morphology and interface.

**Figure 5:**
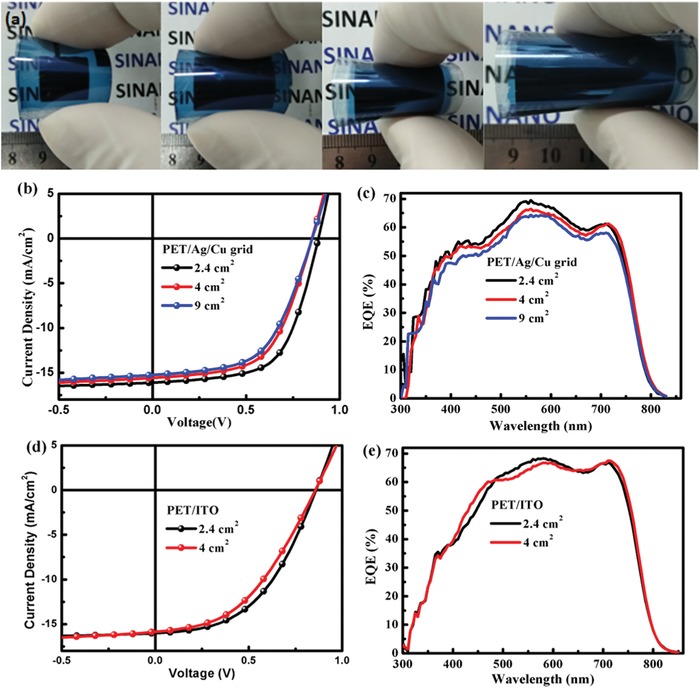
a) Photographs of the large‐area flexible OSCs. b) *J*–*V* characteristics and c) EQE spectra of the large‐area flexible solar cells with PET/Ag/Cu grid electrodes. d) *J*–*V* characteristics and e) EQE spectra of the large‐area flexible solar cells with PET/ITO electrodes.

**Table 2:** Device performance of the large‐area flexible solar cells with PET/Ag/Cu grid and PET/ITO electrodes

Entry	Electrode	Area [cm^2^]	*V*_oc_ [V]	*J*_sc_ [mA cm^−2^]	FF [%]	*R*_s_ [Ω cm^2^]	*R*_sh_ [Ω cm^2^]	PCE [%]	
								Best	Ave.^a)^
13		2.4	0.89	16.12	61	12.4	1060	8.75	8.29 ± 0.14
14	PET/Ag/Cu/E100	4	0.86	15.59	58	16.2	1356	7.79	7.58 ± 0.30
15		9	0.86	15.26	56	28.3	1357	7.35	7.08 ± 0.25
16		2.4	0.86	16.02	48	24.2	1018	6.61	6.44 ± 0.14
17	PET/ITO	4	0.86	15.53	44	33.0	663	5.88	5.76 ± 0.27

^a)^ Average performance calculated over 10–15 individual devices.

In summary, we demonstrated high performance large‐area flexible OSCs using a novel Ag/Cu grid as the flexible transparent conducting electrode. With the modification highly conductive PEDOT:PSS layer (E100) with a suitable thickness, this Ag/Cu/E100 composite electrode showed combination properties of high optical transparency and low sheet resistance, as well as good compatibility with the solution‐processable deposition process of the flexible OSCs. A superior PCE of over 12% for 1 cm^2^ flexible OSCs and a certificated efficiency of 11.45% were obtained, which is currently the performance record. The excellent overall electrical and optical properties of the Ag/Cu grid electrode enabled high performance of the flexible OSCs, and efficiencies of 7.79% and 7.35% for 4 and 9 cm^2^ cell size were achieved with this flexible electrode. In addition, the flexible OSCs exhibited excellent mechanical durability and long‐term stability, and could retain at least 90% of the initial efficiency value after 6 months of storage.

## Experimental Section

*Materials*: PBDB‐T, ITIC, PTB7‐Th, and PC_71_BM were purchased from solarmer Materials Inc, Beijing. PBDB‐TF^70^ and IT‐4F^1^ were synthesized through the route described in the previous references. NF3000‐P and NF3000‐N were provided by Paynergy Tek Inc. Highly conductive PEDOT:PSS (named E100) aqueous was purchased from Heraeus Corporation.

*PET/Ag/Cu Electrode Fabrication*: The PET/Ag/Cu grid electrodes were fabricated through nanoimprinting and electrodeposition according to reported route^47^ with some modifications. Specifically, the fabrication process can be divided into the following five steps. The first step was depositing the UV glue on top of the glass substrate through blading, and then patterning the UV glue films by photolithographing. The Ni master plate was then fabricated through using the pattered UV films as a mask. The second step was pattering the PET substrate: the UV glue was bladed on the top of PET substrate, and then the masklike pattern on PET was formed through imprinting the Ni master plate on the top of UV glue. The third step was filling the Ag nanoink into the trenches through blading and followed by sintering at 150 °C for 15 min. Then the Cu grid was electroplated on the top of the conductive Ag lines for about 5 min with electroplate current of 2 A. Finally, the films were polished to smoothen the surface using the aqueous silica particle solution.

*OSCs Fabrication*: Inverted OSCs with a structure of PET/Ag/Cu/E100/ZnO/active layer/MoO_3_/Al were fabricated on the PET/Ag/Cu electrode. After UV ozone treatment for 10 min, E100 layers with different thicknesses were spin‐coated on the top of the PET/Ag/Cu grid electrodes. The thicknesses of the E100 layers were regulated using different concentrations by diluting the E100 aqueous with different volume ratios of water. Then, the ZnO electron transport layers were deposited on the PET/Ag/Cu/E100 substrate by spin‐coating the ZnO inks at 2500 rpm for 60 s, and annealed at 120 °C for 10 min in the N_2_‐filled glove box. The ZrAcac layer was deposited on top of ZnO film from the 1 mg mL^−1^ ZrAcac solution (in chloroform) at 3000 rpm for 60 s. Then, the active layers (PTB7‐Th:PC_71_BM, PBDB‐T:ITIC, PBDB‐TF:IT‐4F, and NF3000‐P:NF3000‐N) were spin‐coated from a mixed solution of the polymer donors and fullerene or nonfullerene acceptors. For the PTB7‐Th:PC_71_BM devices, the precursor solution composed of PTB7‐Th and PC_71_BM were dissolved in CB, with concentrations of 10 and 15 mg mL^−1^, respectively; the solution also contained 3 vol% DIO as an additive and was stirred at 50 °C for 3 h. Then the mixture solution was spin‐coated on the top of the ZrAcac layer at 1000 rpm for 60 s. For the PBDB‐T:ITIC devices, the precursor solution of PBDB‐T and ITIC was dissolved in CB with concentration of 10 and 10 mg mL^−1^, respectively. For the PBDB‐TF:IT‐4F solar cells, the mixed solution of PBDB‐TF and IT‐4F together with 0.5 vol% DIO was dissolved in CB with concentration of 12 and 12 mg mL^−1^, respectively. Both the PBDB‐T:ITIC and PBDB‐TF:IT‐4F layers were fabricated from spin‐coating at 1500 rpm for 60 s. For the NF3000‐P:NF3000‐N solar cells, 10 mg NF3000‐P, and 10 mg NF3000‐N were dissolved in 1 mL CB at 100 °C for 3 h. The active layer was fabricated through spin‐coating the precursor solution at 2000 rpm for 1 min, and annealed at 120 °C for 5 min. Finally, 10 nm MoO_3_ and 200 nm Al were successively thermally deposited successively on the top of the active layers at a vacuum level below 1 × 10^−4^ Pa. The effective areas of the devices were 1, 2.4, 4, and 9 cm^2^.

*Characterization*: AFM images of the films were measured with the Park XE‐120 microscope using Cr/Au‐coated conducting tips (NSC18, Mikromasch, Tallinn, Estonia). The SEM and cross‐section SEM images of the flexible electrodes and the flexible cells were measured using S4800. The current density–voltage (*J*–*V*) measurements were carried out in a nitrogen glove box with a Keithley 2400 source meter under a Newport solar simulator (100 mW cm^−2^). EQE spectra were measured under simulated 1 sun operation conditions with light from a 150 W tungsten halogen lamp (Osram 64610) as the probe light, a monochromator (Zolix, Omni‐λ300) for selecting the wavelength, and an *I*–*V* converter for recording the response. The light was illuminated on the sample via a small aperture with a radius of 1.5 mm. A calibrated Si cell was used as a reference. The device was kept behind a quartz window in a nitrogen‐filled container. The bending resistance of the devices was measured through manually bending the device surrounding the pillars of different radii for different numbers of cycles and then testing the *J*–*V* characteristics in the glove box. The devices for the long‐term stability test were stored in dark under in the N_2_‐filled glove box and *J*–*V* sweeps were performed after a period of storage under these conditions without further treatment.

## Conflict of Interest

The authors declare no conflict of interest.

## Supporting information

Supplementary
