# Supplementary material for: Efficiency above 12% for 1 cm2 Flexible Organic Solar Cells with Ag/Cu Grid Transparent Conducting Electrode
Source: Adv Sci (Weinh). 2019 Sep 30;6(22):1901490. doi: 10.1002/advs.201901490 (PMC6864593; doi:10.1002/advs.201901490)
Supplement: Supplementary file 1 — Supplementary [file ADVS-6-1901490-s001.pdf]

Copyright WILEY-VCH Verlag GmbH & Co. KGaA, 69469 Weinheim, Germany, 2019.

ADVANCED SCIENCE Open Access

Supporting Information

for *Adv. Sci.,* DOI: 10.1002/advs.201901490

Efficiency above 12% for 1 cm^2 Flexible Organic Solar Cells with Ag/Cu Grid Transparent Conducting Electrode

Yunfei Han, Xiaolian Chen, Junfeng Wei, Guoqi Ji, Chen Wang, Wenchao Zhao, Junqi Lai, Wusong Zha, Zerui Li, Lingpeng Yan, Huiming Gu, Qun Luo, Qi Chen, Liwei Chen, Jianhui Hou, Wenming Su,* and Chang-Qi Ma**

Supporting Information

Efficiency above 12% for 1 cm^2 Flexible Organic Solar Cells with Ag/Cu Grid Transparent Conducting Electrode

Yunfei Han, Xiaolian Chen, Junfeng Wei, Guoqi Ji, Chen Wang, Wenchao Zhao, Junqi Lai, Wusong Zha, Zerui Li, Lingpeng Yan, Huiming Gu, Qun Luo, Qi Chen, Liwei Chen, Jianhui Hou, Wenming Su, Chang-Qi Ma

Yunfei Han, Xiaolian Chen, Junfeng Wei, Guoqi Ji, Chen Wang, Junqi Lai, Wusong Zha, Zerui Li, Lingpeng Yan, Huiming Gu, Dr. Qun Luo, Dr. Qi Chen, Prof. Liwei Chen,

Prof. Wenming Su, Prof. Chang-Qi Ma

School of Nano-Tech and Nano-Bionics, University of Science and Technology of China, Hefei, 230027, P. R. China

Suzhou Institute of Nano-Tech and Nano-Bionics, Chinese Academy of Sciences (CAS),

Collaborative Innovation Center of Suzhou Nano Science and Technology, Suzhou, 215123, P. R. China.

Dr. Wenchao Zhao, Prof. Jianhui Hou

Institute of Chemistry, Chinese Academy of Sciences, Beijing, 100190, P. R. China.

E-mail: qluo2011@sinano.ac.cn; wmsu2008@sinano.ac.cn; cqma2011@sinano.ac.cn

Table S1. The transmittance and the sheet resistance of different electrodes.

Substrate	E100 thickness (nm)	Transmittance at 550 nm (%)	Average visible Transmittance (%) [a]	Sheet resistance (Ω/□)	Φ_{TC} (Ω^{-1})
Glass/ITO	0	89.6	84.5	12.8	2.61×10^{-2}
PET/ITO	0	78.2	79.2	26.8	3.19×10^{-3}
PET/Ag/Cu grid/E100	0	84.8	84.0	0.6	3.20×10^{-1}
PET/Ag/Cu grid /E100	100	81.7	80.5	0.62	2.14×10^{-1}
PET/Ag/Cu grid /E100	215	79.5	78.2	0.76	1.33×10^{-1}
PET/Ag/Cu grid /E100	380	78.7	76.7	0.77	1.18×10^{-1}

a. The average optical transmission is calculated trough dividing the total transmittance in the range of 300 to 1100 nm to the total data number.

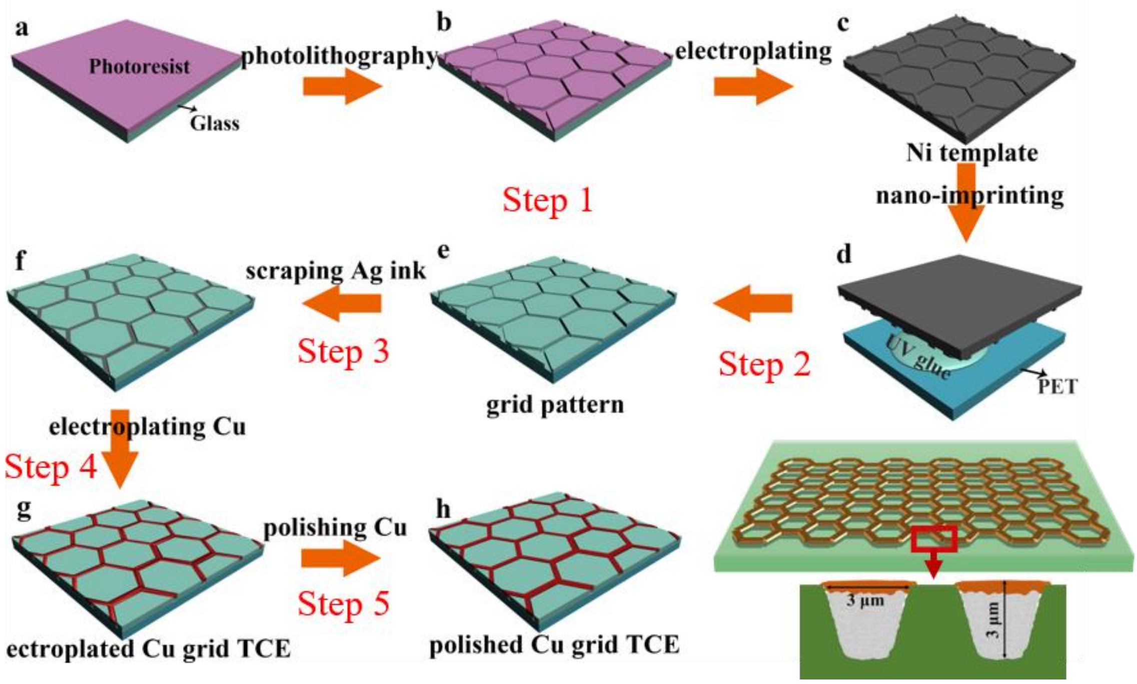

Figure S1. The schematic diagram of the fabrication process of the flexible electrode.

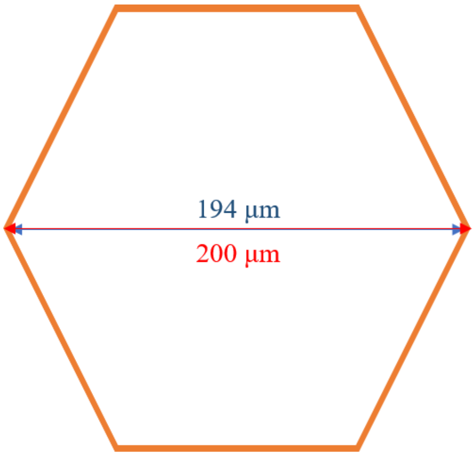

A_s: the area of the small hexagon
A_b: the area of the big hexagon
Because each grid line is co-used by two hexagons,
So, CR (coverage ratio) can be calculated using equation: ***A_b-A_s/2A_b***
As a result,
CR=2.96%.

Figure S2. The coverage of the metal grid on the PET substrate.

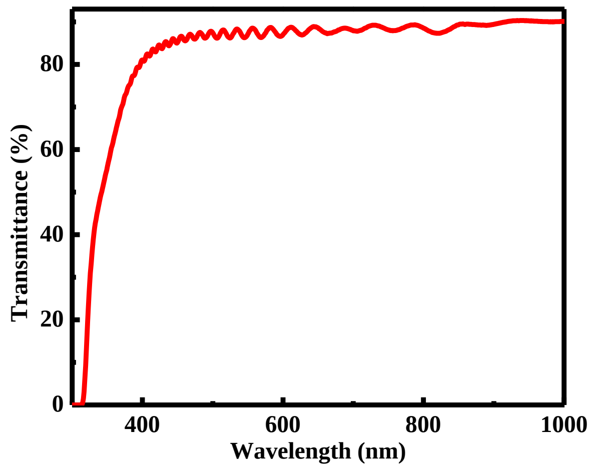

Figure S3. Transmittance spectra of the PET substrate.

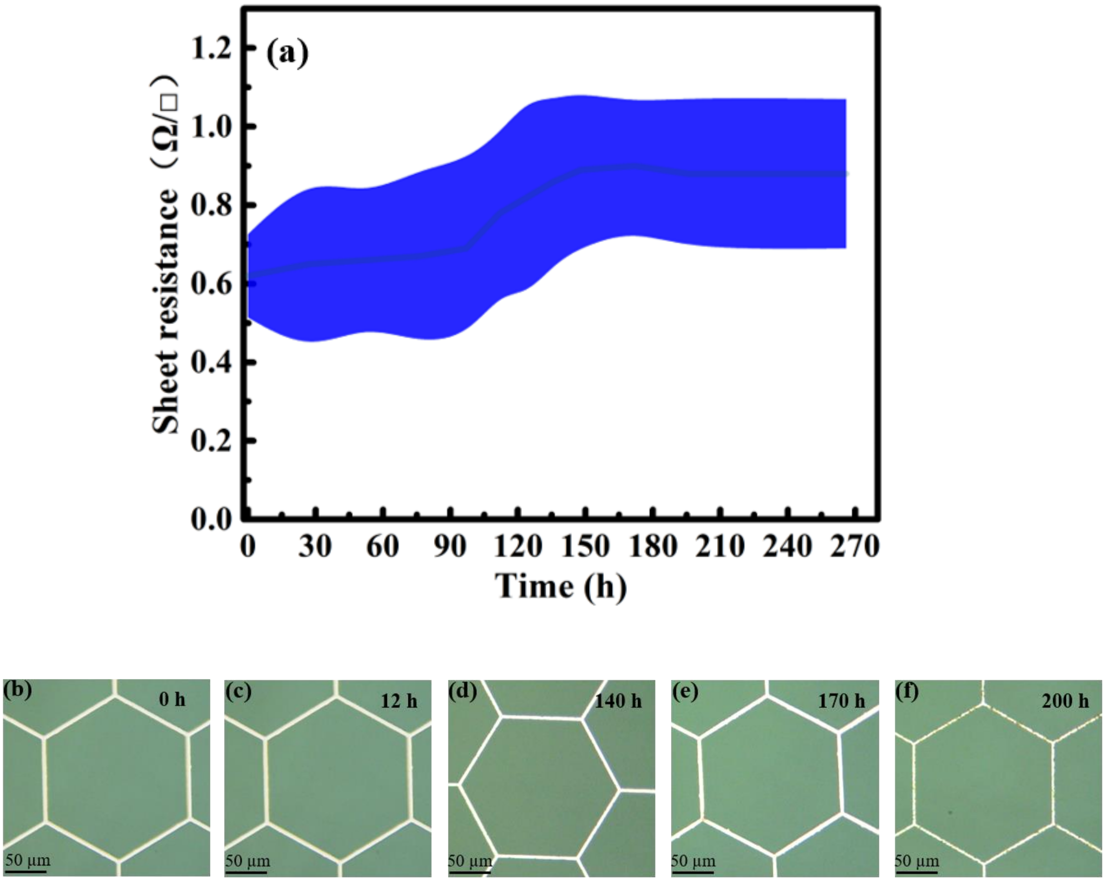

Figure S4. (a) Evolution of the sheet resistance of the flexible electrode during 260 h storage in air. (b) Photographs of the PET/Ag/Cu grid electrode after storage in air for different time.

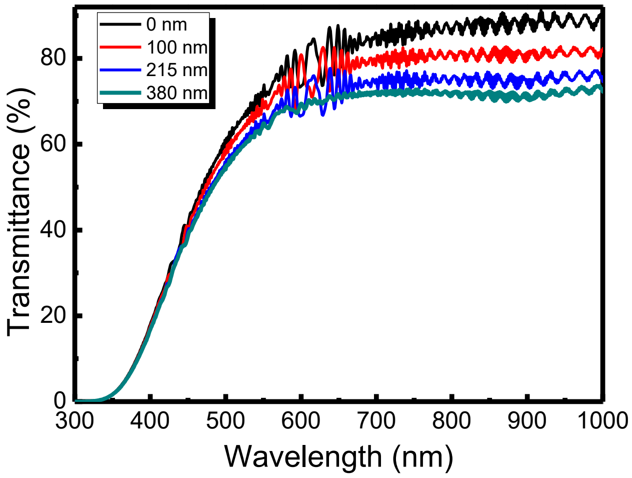

Figure S5. Simulation of the transmittance spectra of the PET/Ag/Cu/E100 composite films with different E100 thicknesses.

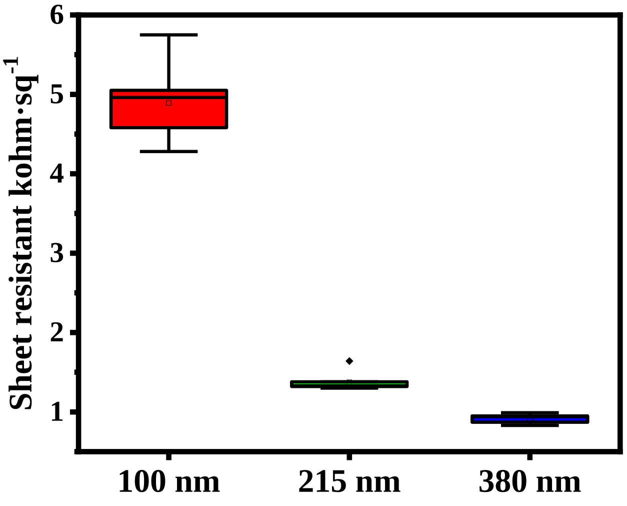

Figure S6. Sheet Resistance of the E100 films that deposited on the glass substrate.

Table S2. The device performance of the 1cm ^{2} PBDB-T: ITIC, PTB7-Th: $PC_{71}BM$, and PBDB-TF:IT-4F flexible solar cells with and w/o ZrAcac layer.

Entry	Active Layer	Cathode Buffer Layer	V_{OC} [V]	J_{SC} [mA/cm^2]	FF [%]	R_S [Ω·cm^2]	R_{sh} [Ω·cm^2]	PCE [%] Best	PCE [%] Ave. [a]
5	PBDB-T: ITIC	ZnO	0.88	15.94	68	9.85	1335.47	9.54	8.83±0.35
7	PBDB-T: ITIC	ZnO/ ZrAcac	0.89	15.48	68	7.77	1427.49	9.37	9.01±0.27
8	PTB7-Th: $PC_{71}BM$	ZnO	0.78	16.23	60	9.24	441.72	7.60	7.12±0.35
9	PTB7-Th: $PC_{71}BM$	ZnO/ZrAcac	0.78	16.58	63	9.91	924.04	8.15	8.01±0.03
10	PBDB-TF: IT-4F	ZnO	0.84	19.63	60	8.22	620.48	9.89	9.33±0.51
11	PBDB-TF: IT-4F	ZnO/ZrAcac	0.84	19.87	67	7.67	897.91	11.18	10.44±0.41

a. Average performance calculated over 10-15 individual devices.

福建省计量科学研究院
国家光伏产业计量测试中心
FUJIAN METROLOGY INSTITUTE
NATIONAL PV INDUSTRY MEASUREMENT AND TESTING CENTER

ilac-MRA　CNAS 中国认可 国际互认 校准 CALIBRATION CNAS L0131

校准证书
CALIBRATION CERTIFICATE

证书编号： (MLY)Q2/19-000505
Certificate No.

委托者 Client	中国科学院苏州纳米技术与纳米仿生研究所/Suzhou Institute of Nano-Tech and Nano-Bionics, Chinese Academy of Sciences (CAS)
委托者地址 Client address	江苏省苏州市工业园区若水路 398 号（398 Ruoshui Road, SEID, SIP, Suzhou, 215123）
器具名称 Instrument	PET/Ag-Cu 基柔性大面积有机太阳能电池(Flexible Organic Solar Cells with Ag/Cu Grid Transparent Conducting Electrode)
制造厂 Manufactory	中国科学院苏州纳米技术与纳米仿生研究所/Suzhou Institute of Nano-Tech and Nano-Bionics, Chinese Academy of Sciences (CAS)
型号规格 Type or Size	1 cm2
器具编号 Instrument number	033-2019-5-5-7
接收日期 Date of Receipt	2019-05-08

福建省计量科学研究院 证书/报告专用章 3501020056403
（机构校准专用章）

批准人： Approved by	[illegible]	批准日期： Date of Approved	2019-05-10
核验员： Checked by	[illegible]	核验日期： Date of Checked	2019-05-10
校准员： Calibrated by	陈彩云	校准日期： Date of Calibrated	2019-05-08

地 址：闽侯经济技术开发区（二期）长龙西路北侧　电 话：0591－87842087　传 真：0591－87811744　邮编：350003　监督电话：0591－87843005
Address. Economic and Technical Development Zone (second phase) chang long road north　Tel. 0591－87842087　Fax. 0591－87811744　Post Code. 350003　Tel.for Supervisor: 0591－87843005

未经本院/中心批准部分采用本证书内容无效。
Partly using this certificate will not be admitted unless allowed by fujian metrology institute

第 1 页/共 6 页
Page of Pages

中国合格评定国家认可委员会（CNAS）实验室认可证书号：No. CNAS L0131

本次校准所依据的技术规范（代号、名称）：
Reference documents for the calibration (code、name)

MLYJZ-Q07-2015 太阳电池 I-V 特性曲线和最大功率校准规范(MLYJZ-Q07-2015 Solar cell I-V characteristic curve and maximum power calibration specification)；IEC 60904-1：2006 光伏器件. 第一部分：光伏电流-电压特性的测量(IEC 60904-1:2006 Photovoltaic devices- Part 1：Measurement of photovoltaic current-voltage characteristics)；JJF 1622-2017 太阳电池校准规范：光电性能(JJF 1622-2017 Calibration Specification for Solar Cells:Photoelectric Properties)；MLYJZ-Q09-2015 太阳电池相对光谱响应度校准规范(MLYJZ-Q09-2015 Solar cell relative spectral response calibration specification)

校准地点及环境条件
Location and environmental condition for the calibration

地点：本院闽侯科研基地 4 号楼 108 室(Room 108, Building 4, MinHou Scientific Research Base)<br>Location		
温度： 25.2 ℃；<br>Temperature	相对湿度： 53 %；<br>Relative Humidity	其它： /<br>Others

本次校准所使用的主要计量标准器具
Main measurement standards used in this calibration

名称<br>Name	编号<br>Number	测量范围<br>Measuring range	准确度等级或最大允差或不确定度<br>Accuracy class or maximum permissible error or Uncertainty of Measurement	溯源机构/证书编号<br>Certificate No.	有效期限<br>Due date
太阳模拟器(Solar Simulator)	2015-006	(400~1100) nm; (800~1200) W/m^2	光谱匹配度(Spectral Match)(400~1100) nm：U_{rel}=8.0% (k=2)；辐照度比(Irradiance Ratio)（辐照度不均匀度、辐照度时间不稳定度(Irradiance Nonuniformity、Irradiance Time Instability)）：U_{rel}=1.2% (k=2)	福建省计量科学研究院(Fujian Metrology Institute)/(MLY)Q2/18-000798	2019-07-23
系统源表(System Sourcemeter)（电子负载(Electronic Load)）	4082810	100 nA~3 A; (0.1~40) V	U_{rel}=0.005%(k=2)	上海市计量测试技术研究院(Shanghai Institute of Metrology and Testing Technology)/2019F11-10-1725983001	2020-02-13
WPVS 单晶硅标准电池(WPVS Monocrystalline Silicon Standard Cell)	015-2014	(300~1200) nm	U_{rel}=1.3% (k=2)	中国计量科学研究院(National Institute of Metrology)/GXtc2019-0450	2020-03-05
自动影像测量仪(Automatic Image Measuring Instrument)	11656	(0~194) mm	U=2 μm (k=2)	福建省计量科学研究院(Fujian Metrology Institute)/(MLY)A2/18-007974	2019-07-26

校准数据/结果：
Data/Results of Calibration

1 标准测试条件(Standard Test Condition) STC：
总辐照度(Total Irradiance)：1000 W/m^2；
被测样品温度(Sample Temperature)：25.0 ℃；
光谱分布(Spectral Distribution)：AM1.5G。

2 STC 下测量数据(Measurement Data under STC)

测量次数 (Test Times)	面积 Area (cm^2)	短路电流 I_{sc}（A）	开路电压 V_{oc} (V)	填充因子 FF (%)	转换效率 η（%）	最大功率 P_{MPP} (W)	最大功率点电压 V_{MPP} (V)	最大功率点电流 I_{MPP} (A)
1	0.9324	0.01823	0.9001	65.64	11.55	0.01077	0.7039	0.01530
2	0.9324	0.01816	0.8963	65.55	11.44	0.01067	0.6928	0.01540
3	0.9324	0.01808	0.8934	65.50	11.35	0.01058	0.6912	0.01530
平均值 (Average Value)	0.9324	0.01816	0.8966	65.56	11.45	0.01067	0.6960	0.01533

3 STC 下电流-电压特性曲线和功率-电压特性曲线(I-V & P-V Curves under STC)

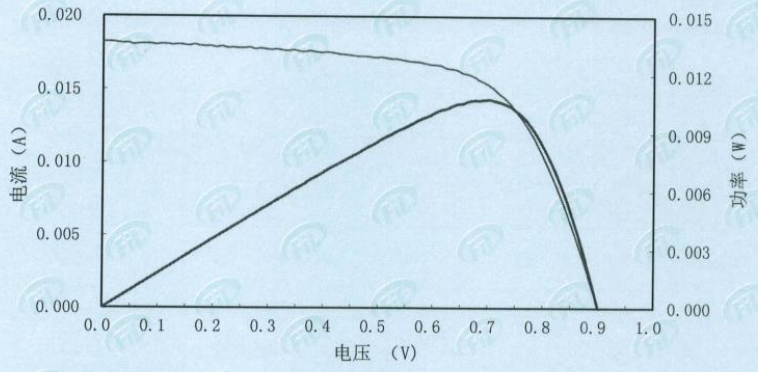

图 1 STC 下被测样品的电流-电压特性曲线和功率-电压特性曲线
(Figure1 I-V and P-V characteristic curves of the measured sample under STC)

4 STC 下被测样品的相对光谱响应和数据(Relative spectral response and data of the Measured Sample under STC)

波长 (Wavelength) /nm	相对光谱响应 (Relative Spectral Response)	波长 (Wavelength)/nm	相对光谱响应 (Relative Spectral Response)	波长 (Wavelength)/nm	相对光谱响应 (Relative Spectral Response)
300	0.0050	580	0.8763	860	0.0100
305	0.0061	585	0.8844	860	0.0081
310	0.0120	590	0.8909	865	0.0064
315	0.0421	595	0.8980	870	0.0046
320	0.0996	600	0.9020	875	0.0031
325	0.1568	605	0.9042	880	0.0019
330	0.1918	610	0.9025	885	0.0016
335	0.2135	615	0.9026	890	0.0015
340	0.2313	620	0.8994	895	0.0012
345	0.2479	625	0.8900	900	0.0010
350	0.2670	630	0.8879	905	0.0008
355	0.2887	635	0.8874	910	0.0009
360	0.3084	640	0.8856	915	0.0005
365	0.3264	645	0.8914	920	0.0005
370	0.3334	650	0.8994	925	0.0009
375	0.3471	655	0.9046	930	0.0009
380	0.3587	660	0.9117	935	0.0006
385	0.3657	665	0.9207	940	0.0004
390	0.3729	670	0.9283	945	0.0005
395	0.3804	675	0.9344	950	0.0005
400	0.3876	680	0.9451	955	0.0006
405	0.3944	685	0.9544	960	0.0009
410	0.4029	690	0.9589	965	0.0007
415	0.4152	695	0.9649	970	0.0006
420	0.4291	700	0.9705	975	0.0004
425	0.4417	705	0.9770	980	0.0002
430	0.4571	710	0.9840	985	0.0004
435	0.4715	715	0.9902	990	0.0002
440	0.4859	720	0.9966	995	0.0002
445	0.5013	725	0.9996	1000	0.0004
450	0.5158	730	0.9999	1005	0.0008
455	0.5309	735	1.0000	1010	0.0007
460	0.5476	740	0.9946	1015	0.0006
465	0.5651	745	0.9889	1020	0.0009
470	0.5790	750	0.9766	1025	0.0006
475	0.5944	755	0.9494	1030	0.0100

波长 (Wavelength) /nm	相对光谱响应 (Relative Spectral Response)	波长 (Wavelength)/nm	相对光谱响应 (Relative Spectral Response)	波长 (Wavelength)/nm	相对光谱响应 (Relative Spectral Response)
480	0.6127	760	0.9165	1035	0.0013
485	0.6297	765	0.8848	1040	0.0015
490	0.6498	770	0.8462	1045	0.0014
495	0.6673	775	0.7786	1050	0.0010
500	0.6850	780	0.6980	1055	0.0010
505	0.7030	785	0.6173	1060	0.0006
510	0.7236	790	0.5391	1065	0.0004
515	0.7414	795	0.4594	1070	0.0004
520	0.7570	800	0.3733	1075	0.0003
525	0.7737	805	0.2945	1080	0.0005
530	0.7871	810	0.2240	1085	0.0003
535	0.8022	815	0.1693	1090	0.0001
540	0.8159	820	0.1186	1095	0.0003
545	0.8292	825	0.0798	1100	0.0006
550	0.8420	830	0.0656	/	/
555	0.8492	835	0.0473	/	/
560	0.8568	840	0.0323	/	/
565	0.8632	845	0.0239	/	/
570	0.8682	850	0.0181	/	/
575	0.8716	855	0.0135	/	/

5 STC 下被测样品的相对光谱响应曲线(Relative spectral response curve of the Measured Sample under STC)

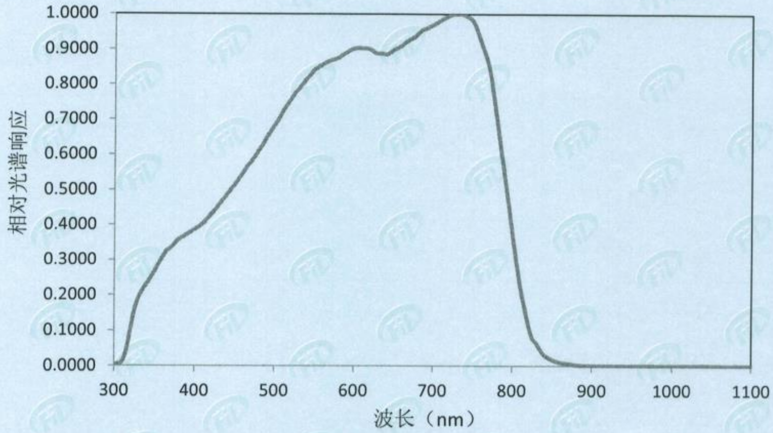

图 2 STC 下被测样品的相对光谱响应曲线

(Figure2 Relative spectral response curve of the Measured Sample under STC)

福建省计量科学研究院/国家光伏产业计量测试中心/校准证书 证书编号： (MLY)Q2/19-000505
CALIBRATION CERTIFICATE OF FUJIAN METROLOGY INSTITUTE/NATIONAL PV INDUSTRY MEASUREMENT AND TESTING CENTER Certificate number

校准结果的不确定度为(Measurement uncertainty of the calibration)：

短路电流(Short-Circuit Current)： U_{rel}= 3.0% (k=2)；

开路电压(Open-Circuit Voltage)： U_{rel}=1.0% (k=2)；

最大功率(Maximum Power)： U_{rel}=3.2% (k=2)；

转换效率(Efficiency)： U_{rel}=3.4% (k=2)；

填充因子(Fill Factor)： U_{rel}=4.4% (k=2)；

相对光谱响应： 300nm~400nm： U_{rel} =2.2 %(k=2)；
400nm~1100nm： U_{rel} =1.8 % (k=2)。

说明：该样品的有效面积为 0.9324 cm^2 (Active Area of the Measured Sample was 0.9324 cm^2)。
Explanation

以下空白
Blank below

本证书提供的结果仅对本次被校的器具有效。
The data are valid only for the instrument(s)

第 6 页/共 6 页
Page of Pages

The certification reports of the *J*-*V* characterization and spectrum response of the flexible solar cell provided by the third party.

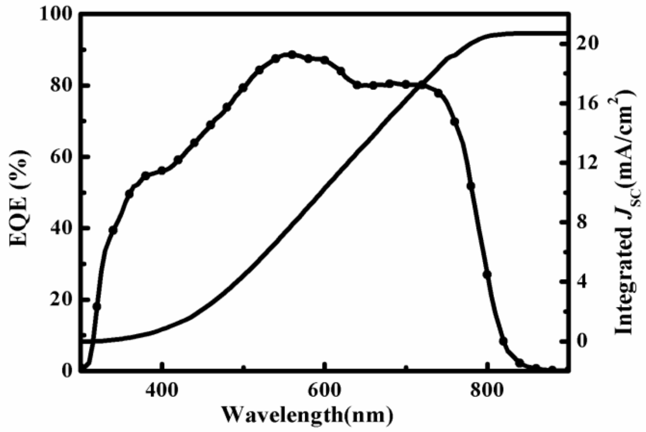

Figure S7. the EQE spectra and integrated current based on the spectrum response provided by the third party.

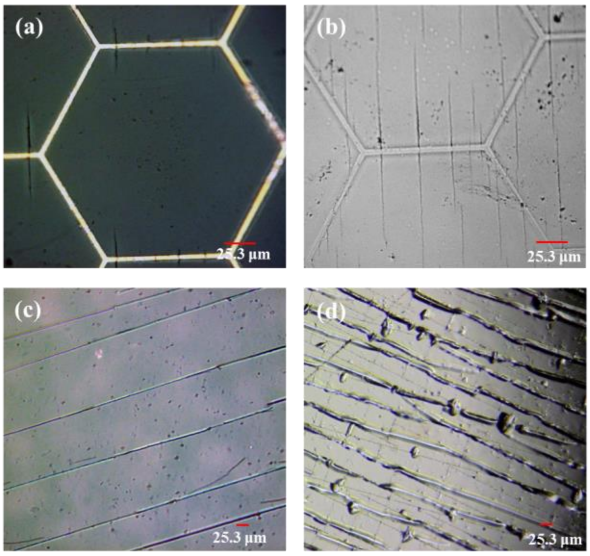

Figure S8. Photograph of (a) the PET/Ag/Cu-grid electrode (b) the PET/Ag/Cu electrode-based device after bending 20 times with radius of 2 mm. (c) Photograph of the PET/ITO electrode, and (d) the PET/ITO electrode-based device after bending 20 times with radius of 3.5 mm.

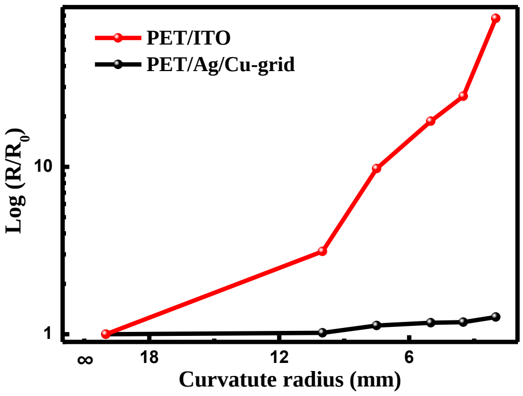

Figure S9. Bending resistance of the PET/ITO and PET/Ag/Cu grid electrodes with different bending radii.

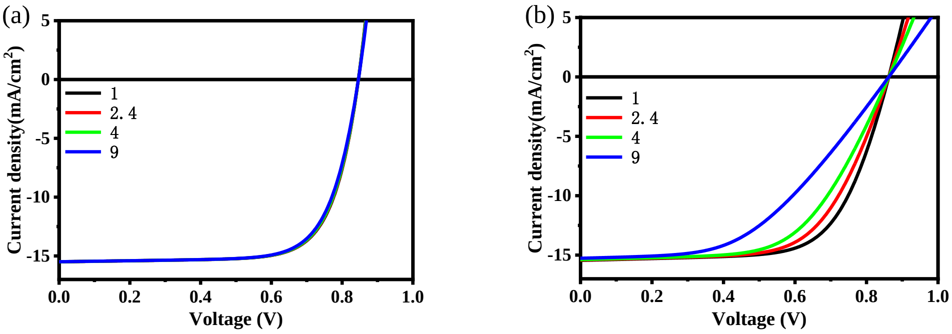

Figure S10. Simulated J-V characteristics of the 1, 2.4, 4, and 9 cm^2 flexible solar cells with (a) PET/Ag/Cu and (b) PET/ITO electrodes.

Table S3. Device performance of the large-area flexible solar cells obtained through simulation.

Electrode	Area [cm^2]	*Voc* [V]	*Jsc* [mA/cm^2]	FF [%]	PCE [%]	R_s [$\Omega\cdot cm^2$]	R_{sh} [$\Omega\cdot cm^2$]
PET/Ag/Cu/E100	1	0.86	15.55	0.73	9.76	0.61	2342
	2.4	0.86	15.55	0.73	9.76	0.72	2342
	4	0.86	15.55	0.72	9.63	0.83	2342
	9	0.86	15.54	0.71	9.48	1.12	2342
PET/ITO	1	0.86	15.44	0.67	8.90	4.73	1231
	2.4	0.86	15.41	0.63	8.40	7.45	1231
	4	0.86	15.37	0.59	7.85	10.55	1231
	9	0.86	15.25	0.48	6.27	20.25	1231
